# Affective and Neural Reactivity to Criticism in Individuals High and Low on Perceived Criticism

**DOI:** 10.1371/journal.pone.0044412

**Published:** 2012-09-11

**Authors:** Jill M. Hooley, Greg Siegle, Staci A. Gruber

**Affiliations:** 1 Department of Psychology, Harvard University, Cambridge, Massachusetts, United States of America; 2 Department of Psychiatry, University of Pittsburgh School of Medicine, Pittsburgh, Pennsylvania, United States of America; 3 Cognitive and Clinical Neuroimaging Core, Brain Imaging Center, McLean Hospital, Harvard Medical School, Belmont, Massachusetts, United States of America; The University of Melbourne, Australia

## Abstract

People who have remitted from depression are at increased risk for relapse if they rate their relatives as being critical of them on a simple self-report measure of Perceived Criticism (PC). To explore neural mechanisms associated with this we used functional magnetic resonance imaging (fMRI) to examine how people with different levels of PC responded to hearing criticism from their own mothers. To maximize variability in affective reactivity, depressed, recovered depressed, and healthy control participants (n = 33) were classified as high or low in PC based on a median split. They were then exposed to personally-relevant critical and praising comments from their mothers. Perceived Criticism levels were unrelated to depression status and to negative mood change after hearing criticism. However, compared to low PC participants, those who scored high on PC showed differential activation in a network of regions associated with emotion reactivity and regulation, including increased amygdala activity and decreased reactions in prefrontal regulatory regions when they heard criticism. This was not the case for praise. Criticism may be a risk factor for relapse because it helps to “train” pathways characteristic of depressive information processing. The Perceived Criticism measure may help identify people who are more susceptible to this vulnerability.

## Introduction

Nobody likes criticism. However, for some people, criticism is especially problematic. For example, people who have had past episodes of depression are much more likely to relapse or show a recurrence of symptoms after recovery if they live in family environments that are characterized by high levels of criticism [1], [2]. The association between criticism and relapse has also been widely replicated for patients suffering from schizophrenia [3]. In addition, other research reports have linked criticism to poor clinical outcomes in patients with such disorders as alcohol dependence, post-traumatic stress disorder, and panic disorder and OCD [4], [5], [6]. Yet, it is unclear how criticism leads to such negative outcomes. To address this question, we used functional magnetic resonance imaging (fMRI) to examine neural response related to criticism in individuals varying in both vulnerability to depression and in perceived criticism.

Much of the evidence documenting a link between criticism and poor clinical outcome has come from research on the expressed emotion construct [7]. Expressed emotion is a measure of the extent to which a relative of a psychiatric patient talks about that patient in a critical, hostile, or emotionally overinvolved manner during a private interview with a researcher. After the interview is competed, a trained rater assesses these elements (the most important of which is criticism) using specific objective criteria. Unfortunately, measuring objective criticism via an expressed emotion interview is very time consuming. However, a growing literature now supports the validity of asking patients to rate (using a 1–10 scale) how critical they believe specific members of their families are of them [8]. Perhaps surprisingly given the simplicity and subjective nature of the measure, patients’ ratings of perceived criticism were strongly predictive of how likely depressed patients were to relapse over the subsequent 9 months; those who rated their spouses as more critical were especially likely to relapse during the follow-up period [9]. Perceived criticism ratings have also been shown to predict relapse, time to relapse, and days abstinent in patients with substance abuse problems [10]. In another study, PC ratings obtained prior to treatment significantly predicted having residual symptoms after a behavioral intervention for patients with anxiety disorders [11]. The originally reported link between PC and relapse in depression has also been replicated [12]. Most recently, research on perceived criticism has been extended to patients with schizophrenia, and PC has been shown to predict an increase in positive symptoms in those at high risk for the development of psychosis [13].

Ratings of perceived criticism may provide a quick and valid way to subjectively assess negative aspects of the psychosocial environment and identify vulnerable people who are at higher risk of worse clinical outcomes. Potentially PC is a proxy for high levels of emotional reactivity or poor regulatory control in the face of affective challenges, either of which could yield poor engagement with psychotherapeutic interventions as well as increased vulnerabilities to be addressed in treatment. There is strong support for this perspective in depression. For example, reactivity to emotional cues in formerly depressed individuals is both elevated [14] and predicts future episodes of depression [15]. When individuals who have recovered from depression are exposed to criticism, they specifically demonstrate decreased reactivity in the dorsolateral prefrontal cortex (DLPFC) compared to never-depressed individuals [16], [17]. Though this region appears to moderate limbic reactivity to emotional cues in healthy individuals [18] its activity and modulatory role is decreased in depressed individuals [19]. Thus, prior research suggests that criticism may be capable of provoking the same types of emotional dysregulation and vulnerability mechanisms that precipitate a transient resumption of the acute state. Such an explanation would suggest that high perceived parental criticism could be associated with a pattern of reactivity more strongly associated with acute psychopathology.

To test this hypothesis, we examined the extent to which exposure to critical comments from one’s own mother was associated with a pattern of activity increasingly implicated in affective psychopathologies such as depression and anxiety, specifically increased limbic reactivity and decreased prefrontal control [20]. We have previously observed both of these phenomena in currently depressed individuals in response to personally relevant emotional stimuli [19], [21]. As PC is associated with increased risk of relapse in depression, these findings provide support for the idea that PC might be associated with differential vulnerability to criticism; specifically, higher levels of reactivity mediated by limbic regions and difficulty with prefrontal control of that reactivity. As there is a broader network of structures also involved in depression, we report on associations throughout the brain as well. However, because our justification for examining other regional involvements is not as strong, these will not be our primary focus. To assess these associations across individuals with a presumably wide range of affective reactivity, we examined reactivity to criticism and to praise in our original sample of healthy and formerly depressed participants [17] as well as in an additional sample of currently depressed participants during functional magnetic resonance imaging (fMRI).

Although not part of the earlier report, we collected perceived criticism ratings from all of our research participants. This afforded us to the opportunity to explore the extent to which PC might play a role in determining neural activity to personally salient critical comments when these are made by the person featured in the PC rating. Our primary hypothesis was that regardless of current or past depression status, higher ratings of perceived criticism would be associated with a vulnerability profile of increased and more long lasting limbic reactivity, specifically localized to the amygdala, as well as decreased and less sustained prefrontal control, specifically localized to the dorsolateral prefrontal cortex. We further hypothesized that this pattern would be unique to criticism and not present in response to praise. This is because the construct of PC is specifically concerned with the processing of criticism.

## Methods

### Participants

Participants were 33 right-handed adult females aged between 19 and 35 years (mean = 25.0, SD = 3.0). Data from 23 of these participants were included in the original Hooley et al. report [17]. Participants were recruited through advertisements in local media. After contacting the research team and completing a brief telephone screening interview, potential participants who appeared likely to meet study entry criteria were invited for a further diagnostic assessment. This was conducted by a trained interviewer using the patient version of the Structured Clinical Interview for DSM-IV (SCID) [22]. An exclusively female sample was chosen in order to minimize heterogeneity in the data due to gender effects and because rates of depression are higher in women than they are in men [23]. Most of our participants were college graduates and all had at least some college experience. All participants provided written informed consent. The study was reviewed and approved by the Committee on the Use of Human Subjects at Harvard University and the Institutional Review Board of McLean Hospital.

Of the 33 participants, 10 met DSM-IV criteria for a current major depressive episode [24]. Another 11 participants had experienced one or more episodes of major depression in the past but were now fully recovered and symptom free. These recovered depressed participants also reported no other current or past Axis I disorders, including all anxiety disorders. A third group was comprised of healthy control participants (n = 12) who were free of current or past psychopathology. The majority of participants (22/33) were not taking any psychoactive medications. Of those who were (6 depressed; 5 recovered depressed) 10 were taking SSRIs and 1 was taking Buspar). Any participant with a history of head injury or neurological problems was excluded from participation.

### Procedure

Subjects participated in two separate research sessions conducted approximately one month apart. During the first visit, they were interviewed with the SCID and completed several self-report questionnaires. These included the Beck Depression Inventory (BDI), the Perceived Criticism Scale (PC), and a trait-form of the Positive and Negative Affect Scale (PANAS). fMRI scans were conducted at the second visit. The BDI was also repeated at this time. Participants were questioned about their current positive and negative mood states before and after the criticism challenge using the PANAS.

### Measures

#### Beck depression inventory

The BDI [25] is a widely used measure of the symptoms of depression. The BDI has 21 items, each of which is rated on a 0–3 scale. A large literature supports the validity of this measure [26].

#### Perceived criticism

Following Hooley and Teasdale [9] perceived criticism was measured with the single question “How critical is your relative of you?” Ratings were made using a 1 (not at all critical) to 10 (very critical) scale. All participants were asked to rate their mothers. Previous research suggests that the PC scale has good predictive validity [27] is not correlated with current symptoms of depression or anxiety, and has high (*r* = .75) test-retest reliability over short (two weeks) and longer (20 weeks) intervals [6], [9]. PC ratings are also significantly correlated with lower ratings of marital satisfaction as well as higher ratings of EE [9], [28], [29]. Recent research has further confirmed that PC ratings reflect perceptions of harsh or hurtful (destructive) criticism as opposed to criticism that is regarded as more constructive [30], [31].

#### Positive and negative affect schedule

The Positive and Negative Affect Scale (PANAS; [32] contains ten positive (e.g., interested, proud) and 10 negative (e.g., ashamed, irritable) mood descriptors. Each item is rated on a 1–5 scale (1 = very slightly or not at all; 5 = extremely). Scores are then summed to give a total for positive affect and for negative affect. The positive and negative PANAS scales have good internal consistency, reliability and validity [32].

### Criticism Challenge

Details of the criticism challenge and the efforts made to ensure the protection of research participants are provided elsewhere [17]. Briefly, while inside the scanner, we exposed participants to comments made by their own mothers. These comments were recorded during a telephone conversation with the mothers in which they were asked to provide personally criticizing comments about their own daughters. Mothers were also asked to make praising comments and these were also recorded. All mothers were free to choose the content of each criticizing or praising statement, each of which lasted 30 seconds. Mothers were asked to begin all critical remarks in a standard way (e.g., “Stacey, one thing that really bothers me about you is…”). Praising remarks began, “Stacey, one thing I really like about you is….” If mothers made a mistake when they were making a specific comment or if the length of the comment was too long or too short, we allowed them to make as many attempts as they wanted to get the comment to sound natural and be of the right length. After the recordings had been made, we asked mothers to refrain from talking to their daughters about the content of their conversations with the research team until after the scanning had been completed. All mothers provided oral consent to these procedures. Consent was documented on the recording itself, at the beginning of the conversation, and this method of consent was approved by the IRB.

All recorded telephone conversations with mothers were subsequently edited to extract the stimulus comments that were to be used in the study from the general conversational content. The stimulus comments were then transferred to CD. During fMRI, participants heard the comments over non-ferrous, gradient damping headphones, in the context of a blocked design. The order of presentation of comments (criticism or praise first) was randomly determined for each participant.

### Presentation of Stimuli

All participants heard both critical and praising comments. Two comments of each type (e.g., criticism, praise) were presented within a given scanning epoch. Each epoch, which lasted for 2∶31, began with a 30 second rest period. This was followed by 30 seconds of commentary, another rest period, another 30 seconds of the same type of commentary, and then another rest period. Thus, in each epoch, participants heard two 30 second segments of each type of commentary. There was no commingling of comment type in the same scan epoch; participants heard either two critical or two praising comments from their mothers. Each individual comment was heard only once and participants did not hear any of the recorded maternal comments prior to the scanning.

### Imaging Methods

Functional images were acquired on a 1.5 Tesla GE LX MRI scanner equipped using a birdcage quadrature RF head coil (TR = 3 sec, TE = 40 msec, flip angle = 90 degrees). Images were collected over 20 coronal slices with a 20 cm field of view and a 64×64 acquisition matrix (in-plane resolution = 3.125×3.125×7 mm). For each 150 second scan, 50 echoplanar images were collected each consisting of five alternating 30-second control/task periods. Matched T1-weighted high-resolution images were also collected for every participant. Head motion was minimized with foam padding and a stabilization strap across the forehead.

Following time-slice correction, functional data were motion corrected using AFNI’s [33] 3dVolReg using the first image as a reference, detrended, and outliers >1.5IRQ from the 25^th^ or 75^th^ percentile were Windzorized. Data were temporally smoothed using a 7 scan Gaussian filter, warped to the Colin-27 Montreal Neurological Institute (MNI) template using AIR’s 32 parameter non-linear warping algorithm and spatially smoothed using a 6 mm FWHM filter.

To understand moderation of criticism effects by PC, we used a criticism/rest × PC (high/low) × scan-within-blocks (10 levels from 0–30 s) ANOVA in which subject was a random factor, criticism and scan were within-subjects factors and group was a between subjects factor. In this way, regions that had a different magnitude of hemodynamic response would reveal main effects of group or condition, and regions with differently shaped responses as a function of condition or group would have interactions of group or condition with scan. To control Type I error, regions were preserved with voxelwise significance of *p*<.005 subject to empirically determined contiguity thresholds using Monte Carlo Simulations (AFNI’s 3dAlphaSim) based on the spatial autocorrelation of effect maps, with autocorrelation computed using AFNI’s 3dFWHM. To capture extended processing we specifically examined effects in resulting regions in an *a priori* temporal interval of 21–30 seconds following the onset of criticism compared to a pre-stimulus baseline.

To understand the extent to which variance in observed effects could have been a function of diagnosis, effects in regions of interest were examined after covarying dummy-coded diagnosis. Finally, to understand the extent to which observed effects were unique to criticism, effect magnitudes for praise were also examined in the same regions.

## Results

### Diagnosis: Differences Associated with Perceived Criticism

A one-way ANOVA revealed that participants’ ratings of how critical their mothers were (perceived criticism) did not differ significantly across the currently depressed, previously depressed, and control groups, *F*(2,30) = 0.32, *p* = .73. Similarly, and consistent with the broader literature, there was also no correlation between PC ratings and scores on the BDI, either at the baseline assessment, *r*(33) = −.15, *p* = .40 or when BDI was measured on the day of the scan, *r*(33) = .03, *p* = .87, as well as self reports of PANAS trait positive mood, *r*(33) = .00, *p = *.98, and PANAS trait negative mood, *r*(33) =  −.10, *p* = .57. We therefore combined the three diagnostic groups for all subsequent analyses.

Across all participants, the mean PC rating that was assigned to mothers was 4.44 (SD = 2.63; range 1–9). There is no *a priori* clinical threshold for PC. Therefore, to yield maximal power to analyze group differences given our relatively low N, and to aid interpretation of findings, we used a median split (median = 4.0) to divide participants into 2 groups of high (n = 17) versus low (n = 16) PC scorers. We then compared participants’ reactions to hearing critical comments from their mothers. As shown in Table 1, the frequency of high PC was evenly distributed across all three diagnostic groups, χ^2^ = 0.61, *p* = .97.

**Table 1:** Perceived Criticism and Diagnostic Group.

Diagnostic Group		Perceived Criticism		Total
		Low	High	
Group	Control	6	6	12
	Recovered Depressed	5	6	11
	Depressed	5	5	10
Total		16	17	33

Note: The frequency of high PC was evenly distributed across the three diagnostic groups, χ^2^ = 0.61, *p* = .97.

### Self-report: Differences in Negative Affect Associated with PC

Participants provided mood ratings (assessed using the PANAS) before and after they heard the critical comments. A PC group (high/low PC) × time (before/after hearing criticism) ANOVA on negative mood suggested that although there were no PC group differences in change in negative mood (PC × time *F*(1, 31) = .11, *p*>.5), negative mood increased in both groups after hearing criticism (negative mood before = 13.36; negative mood after = 15.76, time main effect, *F*(1, 31) = 8.90, *p* = .006, partial η^2^ = .22). Immediately upon exiting the scanner, we also asked participants to rate, on a 1 (low) −10 (high) scale, how negative and how upsetting the critical comments they had heard were. High and low PC scorers rated their mothers’ critical comments as comparably negative (high PC = 6.59; low PC = 6.59, *t*(31) = −.01, *p* = .99. They were also similarly upset by the remarks (high PC = 5.12; low PC = 5.91, *t*(31) = − 0. 96, *p* = .34. Interestingly, however, participants who scored higher on PC rated their mothers’ criticisms as more familiar (i.e., something they had heard before) than low PC participants did (high PC = 8.10; low PC = 5.59, *t*(31) = 2.90, *p* = .009, *d* = 1.04).

### fMRI: Reactions to Hearing Criticism

**Figure 1:**
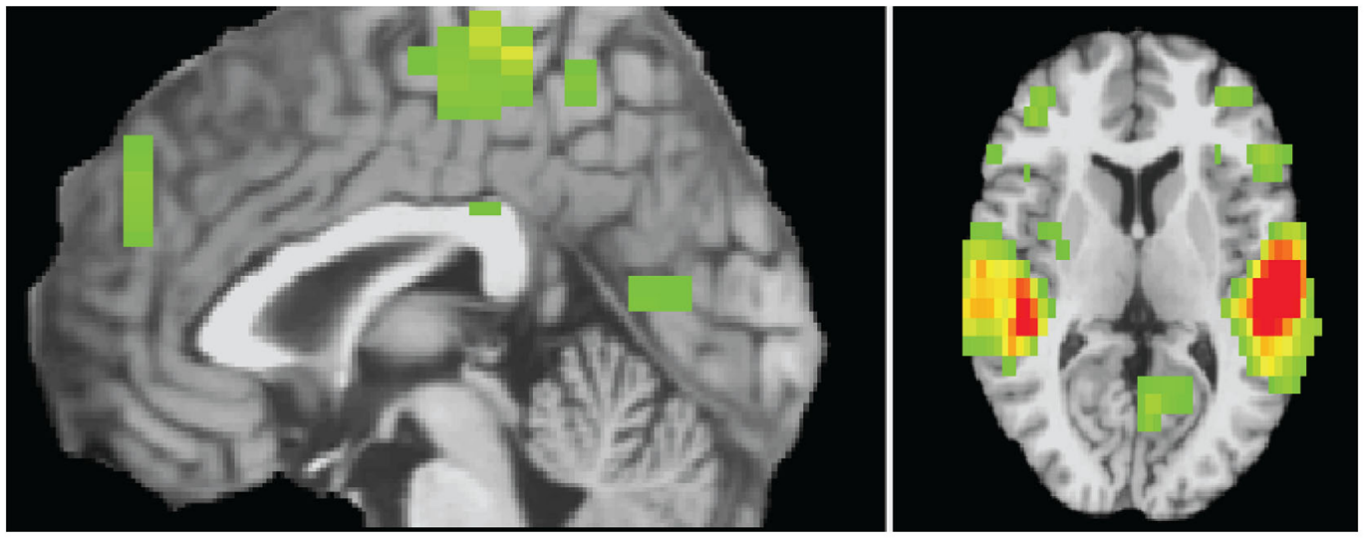
Empirically determined regions displaying condition (criticism/rest) × scan interactions, *p*<.005, 30 voxels contiguity.

Imaging data were available for 26 participants. As was the case with the full sample, there was again no association between diagnosis and PC in this subgroup, *X* ^2^ = 1.47, *p* = .48. As shown in Figure 1, a PC group (high/low PC) × condition (criticism/rest) × scan ANOVA revealed that hearing criticism was associated with increased activity compared to rest in a widespread cortical/subcortical network, particularly in the auditory cortex. This latter result is unsurprising given that the task is verbal. No regions displayed decreased reactivity to criticism compared to rest.

### fMRI: Differences in Response to Criticism Associated with PC

**Figure 2:**
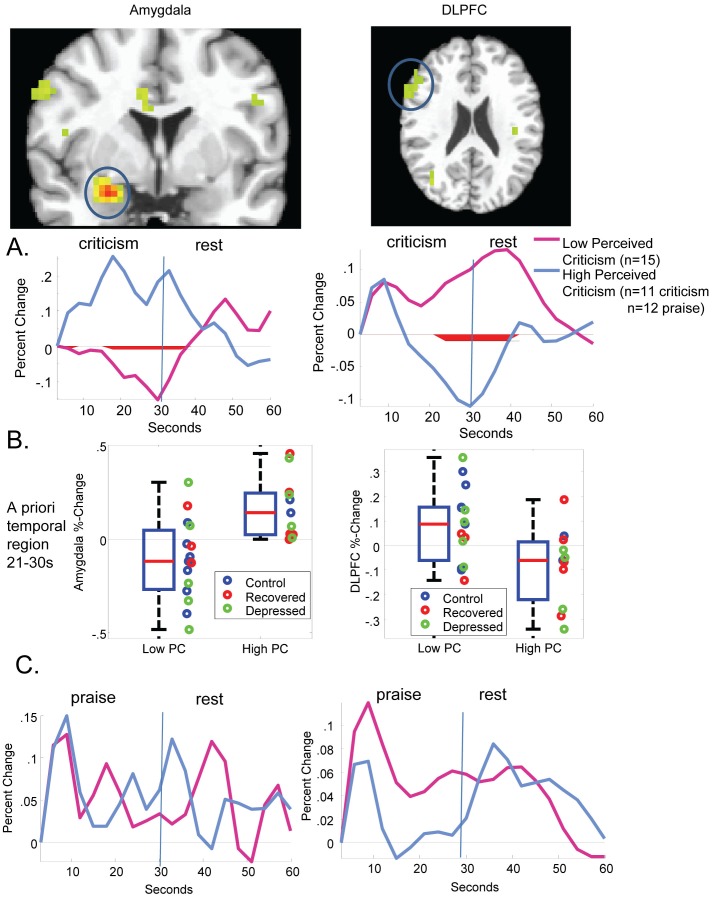
Empirically determined regions within *a priori* specified structures displaying condition (criticism/rest) × **PC** × **scan interactions, p<.005, 12 voxels contiguity.** Shown for (A) criticism (B) criticism in the a priori temporal period of 21–30 seconds following the onset of criticism, and (C) praise.

As shown in Table S1, a PC group (high/low PC) × condition (criticism/rest) × scan ANOVA revealed 19 ROIs spanning an expected network of cortical and subcortical regions characterized by group × condition × scan interactions, *p*<.005 and 12 voxels contiguity. Of particular note, a PC × scan effect in the amygdala survived mixed effects analysis using an AR1 covariance structure, *F*(19,354.6) = 1.89, *p = *.01. As shown in Figure 2, there was a trend towards increased activity in the right amygdala in the high compared to the low PC group that began early in the criticism period, 6.00 to 9.00 s: *t*(24) = 1.96, *p = *0.06, difference in %-change (D) = 0.12, *d = *0.78. This pattern increased as the criticism progressed, was maintained for the duration of the criticism, and continued into the rest period 18.00 to 39.00 s: *t*(24) = 3.99, *p*<0.005, D = 0.25%, *d* = 1.58. Relative to the low PC group, the high PC group also showed decreased and less sustained activity in the right dorsolateral prefrontal cortex in response to criticism, mixed effects *F*(19,411) = 2.11, *p* = . 004, which lasted into the rest condition 24.00 to 42.00 s: *t*(24) = − 2.85, *p = *0.01, D = − 0.17, *d = *− 1.13. Effects in the *a priori* temporal region at 21–30 seconds following the onset of criticism (coded as area under the curve compared to the pre-criticism baseline; AUC) are shown in Figure 2B and Table 2.

**Table 2:** fMRI Effects 21–30 Seconds Following the Onset of Criticism.

Region	Effect	Statistic	*p*	ES
Amygdala	High v. Low PC	t(24) = 3.56	.002	d = 1.36
DLPFC	High v. Low PC	t(24) = 2.71	.01	d = 1.03
Effects of regressions including group status				
Amygdala	PC	F(1,24) = 12.7	.002	R^2^ = .34
Amygdala	Diagnosis	F(2,23) = 1.05	.36	R^2^ = .08
Amygdala	PC above and beyond diagnosis	F Δ(1,22) = 9.3	.006	ΔR^2^ = .27
Amygdala	PC × diagnosis	FΔ(2,20) = .45	.66	ΔR^2^ = .03
DLPFC	PC	F(1,24) = 7.39	.01	R^2^ = .24
DLPFC	Diagnosis	F(2,23) = 1.97	.32	R^2^ = .09
DLPFC	PC above and beyond diagnosis	FΔ(1,22) = 5.04	.03	ΔR^2^ = .17
DLPFC	PC × diagnosis	FΔ(2,20) = 1.4	.29	ΔR^2^ = .09

### fMRI: Independence of Amygdala and DLPFC Associations with PC

Associations of amygdala and DLPFC activity with perceived criticism were largely independently associated with PC. That is, a simultaneous logistic regression on PC revealed independent effects of amygdala AUC, *B* = 8.95, Wald(1) = 4.89, *p* = .02, Exp(B) = 7754.8, and DLPFC AUC, *B* = −7.07, Wald(1) = 3.92, *p = *.05, Exp(B) = .001; 22/26 (84%; 13/15 low criticism, 9/11 high criticism) correct classification. These features were also were independent from each other, and did not qualify each other’s effects on PC. Specifically, a hierarchical regression on DLPFC AUC in which the AUC of amygdala activity was entered on step 1, PC on step 2, and an amygdala × PC interaction on step 3 revealed a minimal association of amygdala with DLPFC activity, *R^2^* = .07, *F*(1,24) = 1.9, *p = *.18. Moreover, the association of PC with DLPFC was significant even after accounting for amygdala activity, *ΔR^2^* = .16, *F Δ(*1,23) = 4.9, *p = *.04. There was also no amygdala × PC interaction on DLPFC activity, Δ*R^2^* = .02, *F* Δ(1,22) = 0.6, *p = *.44.

### fMRI: Additive Effects of PC and Depression Status

**Figure 3:**
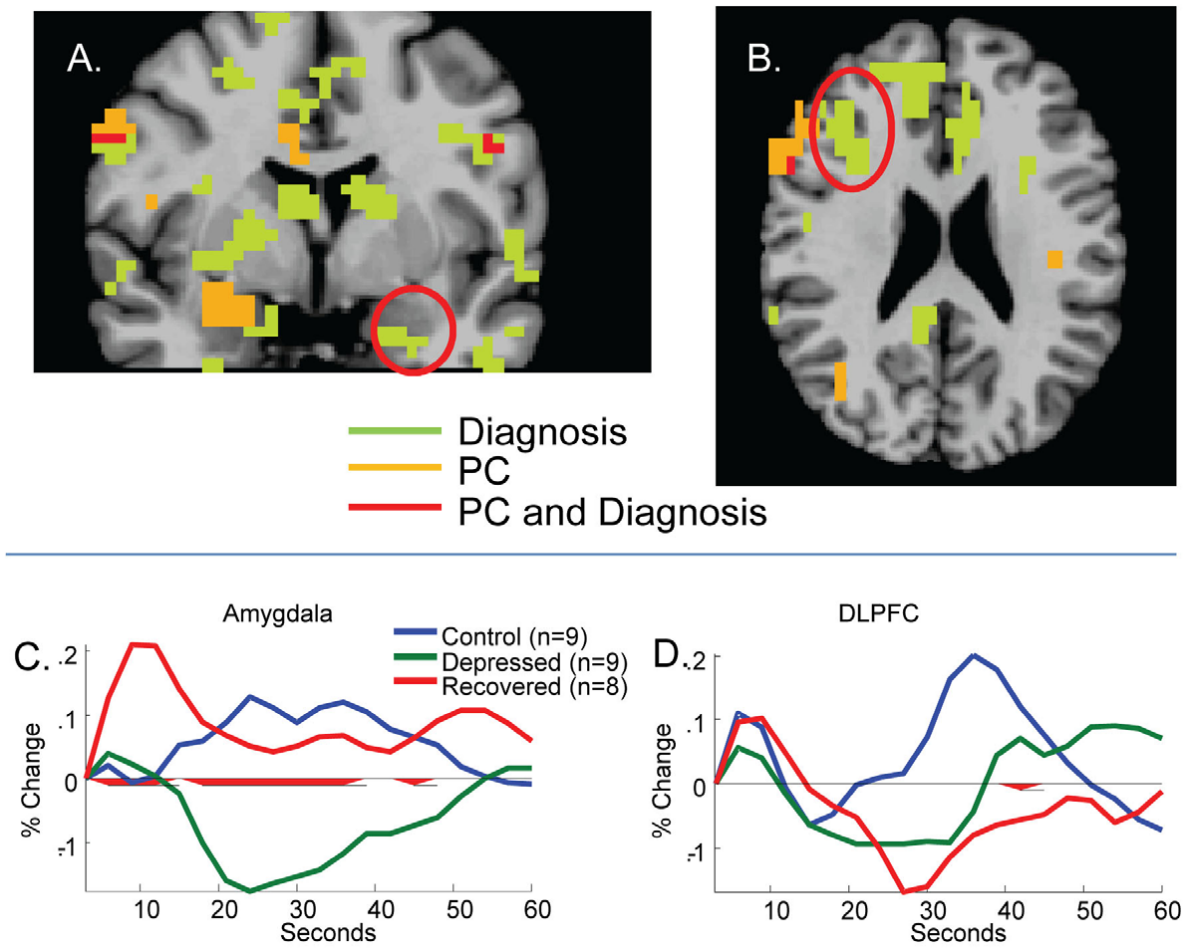
Brain regions associated with diagnosis × condition × scan (green) and PC × condition × scan (orange) interactions and their overlap (red) in (A) amygdala and (B) DLPFC. As shown in the figure, these effects were largely divergent. Time-series showing replication of the Hooley et al. [17] effects in recovered depressed participants from areas significant in the diagnosis × condition × scan analysis are shown for amygdala (C) and DLPFC (D).

As shown in Figure 3, a voxelwise diagnosis × condition (criticism/rest) × scan ANOVA yielded amygdala and DLPFC regions associated with diagnosis, *p*<.005, 15 voxels contiguity, replicating the Hooley et al. [17] result showing increased amygdala and decreased DLPFC activity in recovered depressed compared to healthy individuals. That said, the obtained amygdala and DLPFC regions were generally non-overlapping with the amygdala and DLPFC regions obtained in the PC analysis. In other words, the spatial extent of amygdala and DLPFC reactivity to criticism was effectively additive in association with diagnosis and PC.

Moreover, as shown in Figure 2B and Table 2, diagnosis did not moderate the PC effect in regions obtained in the PC analysis. That is, variance in AUC associated with PC group did not decrease appreciably when covaried for diagnosis, even in our small sample. Rather, in a hierarchical regression on amygdala activity in the *a priori* 21–30 s period, in which dummy-coded diagnosis was entered on the first step, *R* ^2^ = . 08, *F*(2,23) = 1.05, *p = *.36, PC explained an additional *ΔR^2^* = 27.3% of the variation, *F*(1,22) = 9.3, *p* = .006, total *R^2^* = .36. In a similarly structured hierarchical regression on DLPFC activity, diagnosis explained, *R^2^* = .09, *F*(2,23) = 1.20, *p = *.32, and PC explained an additional Δ*R^2^* = 16.9% of the variation, *F*(1,22) = 5.0, *p = *.04, total *R^2^* = .26. The amygdala and DLPFC regions were also not characterized by diagnosis × PC × condition × scan interactions.

### fMRI: Specificity of PC-related Differences to Criticism

In the amygdala, there was a significant mixed effects scan × PC × valence (praise/criticism) interaction, *F*(19, 683) = 1.96, *p* = .009, such that observed effects for criticism were not replicated for praise in the amygdala, mixed effects PC × scan interaction F(19,400.02) = 1.28, *p = *.19. For the DLPFC, there was a marginally significant scan × perceived criticism × valence effect, *F*(19,679.0) = 1.49, *p* = . 08 and significant perceived criticism × valence effect, *F*(1, 429.4) = 19.9, *p*<.0005 with a larger PC differential response in the criticism condition than in the praise condition. As was found for the amygdala, there was no mixed effects PC × scan interaction *F*(19, 437.305) = 1.11, *p = *.34 for praise in the DLPFC. As shown in Figure 2C, there were no significant differences (*p*<.05) between the high and low PC groups at any scan along the waveforms associated with reactivity to positive information, suggesting that the effects described above were specific to criticism.

## Discussion

We examined the extent to which affective and neural responses to maternal criticism were associated with how critical participants rated their mothers as being on a self-report measure of perceived criticism. Data suggested that perceptions of increased maternal criticism were not associated with increased negative affect or greater upset after hearing critical remarks from the mother. However, participants who rated their mothers as more critical showed decreased prefrontal control activity and increased and more prolonged limbic reactions in response to acute criticism. Importantly, activity in these two regions was largely independent, suggesting multiple vulnerabilities.

Elsewhere, we have demonstrated that relative to controls, recovered depressed participants demonstrate activation within the amygdala in response to criticism and fail to activate DPLFC and anterior cingulate cortex (ACC) [17]. In the current study, we found no evidence that ACC activity was moderated by perceived criticism. This is consistent with the idea that the differences in ACC activation are specifically related to depression and vulnerability to depression, as is suggested by current models [34]. However, the affective and neural reactivity we observed in amygdala and DLPFC in this study was not a function of diagnosis. This may, at first glance, seem surprising. Further analyses revealed that the diagnosis-related activation that occurs in regions of the DLPFC and amygdala are different from those that are activated during criticism in high versus low PC individuals, yielding a picture of additive effects of diagnosis and PC. We also found no activation in these areas during exposure to praise in the high versus low PC groups. Overall, the observed patterns of reactivity that we report here may reflect specific vulnerabilities to the effects of criticism that are independent of those associated with diagnosis.

The observation of increased amygdala reactivity and decreased DLPFC activity during criticism are interesting for several reasons. Both are key structures in emotion-attention networks in the brain. Though the DLPFC plays a role in emotion regulation through initiating inhibition of neural mechanisms of emotion including the amygdala [35], [36], [37], [38], we did not find evidence for systemic moderation. Rather, our data were more consistent with the idea of two vulnerabilities with additive effects; increased limbic reactivity and decreased cognitive control were both associated with high PC, yielding the linearly highest level of PC among those with both features. Abnormalities in amygdala and DLPFC activity have been well demonstrated in depression, especially during tasks that involve emotion processing [19], [39]. These abnormalities are also independent of mood state [16], [17]. The finding of independent associations here suggests that there could be multiple pathways to or possibly from high PC.

PC is a relationship-specific measure. Accordingly, a given person could provide a high or a low PC rating, depending on the relationship that is being assessed (e.g., how critical is your mother of you?; how critical is your father of you?). It is also important to note that PC is not simply a measure of negativity or neuroticism, and that PC in this study was not correlated with high trait negative mood or low trait positive mood assessed using the PANAS. PC is thought to predict relapse in depression because it provides a measure of “how much criticism is getting through to the patient” within a family context regardless of what the objectively assessed “reality” might be [9]. Some people may live in genuinely critical family environments and their high PC ratings may accurately reflect this. Others, however, may be more sensitive and “thin skinned” feeling criticized in the absence of genuine criticism. People who have predispositions to strong automatic reactions to emotional information (increased amygdala reactivity) or who fail to recruit voluntary executive control in the face of emotion (decreased DLPFC function) might well fall into this category. Having both vulnerabilities could lead to even stronger feelings of being criticized and even higher PC ratings. It is also possible that over time, continued exposure to high levels of family criticism could lead to increased sensitivity to emotion via sensitization or through a more passive pattern of failure to engage regulatory resources (or both) in a manner more akin to learned hopelessness. Future work is clearly needed to identify individual or interpersonal factors that might predispose individuals to one or the other such pattern.

Strengths of our study include the use of a highly naturalistic paradigm involving mothers making critical and praising comments that were personally relevant for our participants. It is also important to note that we asked all mothers to begin their comments in a standardized way. Critical comments all began with the phrase “One thing that bothers me about you ….” All participants were therefore aware that they were being criticized within the first few seconds of each comment. However, the criticism then continued and was elaborated for the full 30 s duration of each remark, leading us to select the later stages of the comments as an *a priori* temporal period of interest.

Although intriguing, results from the present study should be interpreted in light of several limitations. We do not know how objectively critical the mothers who provided the critical comments actually were. Also, small samples within each of the diagnostic subgroups may have prevented detection of differentiation in PC associated with diagnosis at the neural level. That said, as our inclusion of multiple diagnostic groups was used primarily to increase power for the PC analysis, diagnosis effects were unlikely to have obscured the PC findings, with the possible caveat that there were very few high PC control participants. Though all of the high-PC control participants showed the expected pattern, it remains possible that controls with high PC show differential brain activity relative to individuals with high PC who have a depression history. In addition, due to the cross-sectional nature of this study, no causal inferences can be made. It remains for future research to explore whether PC can predict the onset of disorders in currently healthy people. Finally, a greater understanding of the PC construct itself is very much needed. Future studies should examine whether PC is associated with attentional or information processing biases. The possible links between PC and early childhood adversity also warrant consideration.

These limitations notwithstanding, our general findings linking higher perceptions of criticism to higher levels of limbic reactivity and decreased prefrontal control are exciting. Furthermore, because they are independent of psychiatric history, our results provide potentially important bridges between the social neuroscience and clinical literatures. More specifically they raise the possibility that high ratings of perceived criticism could be used to identify people who have problems in emotion or cognitive control networks and who are thus differentially vulnerable to the detrimental effects of psychosocial stress [40].

In the future, it would be especially informative to explore whether PC is associated with differences in performance on cognitive processing tasks designed to examine orienting and executive control when responding to negative stimuli. Although highly speculative at this time, it is possible that people who feel more highly criticized may have more difficulty inhibiting socially relevant negative information. If PC is linked to problems with automatic emotion regulation, this would provide a possible explanation for why PC is associated with a broad range of unfavorable clinical outcomes.

As we have noted earlier, PC is known to predict relapse in depression [2], [12] as well as negative clinical outcomes in several other disorders, although such associations are not invariably found [41]. Our data suggest that there may be multiple identifiable routes to vulnerability and that these routes may also provide targets for intervention. Activity in some of the regions we have identified have already been shown to be predictive of response to specific treatments such as Cognitive Therapy [42]. If high ratings of perceived criticism can be used to identify behavioral subgroups with high amygdala and low DLPFC reactivity to naturally occurring psychosocial stressors, it is possible that PC ratings (which are very simple and quick to obtain) could be used to select the people who might benefit most from cognitively-based interventions.

## Supporting Information

Table S1 Regions displaying condition × PC × scan interactions, *p*<.005, 12 voxels contiguity in the whole brain. Separate ROI’s are reported for regions that intersect a structure. For example, if regions intersect the posterior cingulate in two places but do not touch, 2 ROIs are reported each with a different centroid within the posterior cingulate. To aid interpretability only sub-regions with >12 voxels are reported unless the only intersecting sub-region was <12 voxels in which case it was reported, as it was contiguous with a larger region that passed the brain-wise contiguity threshold. Talairach coordinates are reported. The amygdala and DLPFC illustrated in Table 2 and Figure 3 are highlighted.
(DOC)
