# Supplementary material for: Affective and Neural Reactivity to Criticism in Individuals High and Low on Perceived Criticism
Source: PLoS One. 2012 Sep 11;7(9):e44412. doi: 10.1371/journal.pone.0044412 (PMC3439418; doi:10.1371/journal.pone.0044412)
Supplement: Table S1 — Regions displaying condition × PC × scan interactions, p<.005, 12 voxels contiguity in the whole brain. Separate ROI’s are reported for regions that intersect a structure. For example, if regions intersect the posterior cingulate in two places but do not touch, 2 ROIs are reported each with a different centroid within the posterior cingulate. To aid interpretability only sub-regions with >12 voxels are reported unless the only intersecting sub-region was <12 voxels in which case it was reported, as it was contiguous with a larger region that passed the brain-wise contiguity threshold. Talairach coordinates are reported. The amygdala and DLPFC illustrated in Table 2 and Figure 3 are highlighted. (DOC) [file pone.0044412.s001.doc]

**Table S1: Regions Displaying Condition x PC x Scan interactions *p* < .005, 12 voxels Contiguity in the Whole Brain.**

**# mm avg max X Y Z**

*Posterior Cingulate*

1 65 0.02 0.07 14 -44 8

2 262 0.05 0.15 -20 -62 6

*Subcallosal Gyrus*

1 346 0.04 0.15 20 12 -13

*Transverse Temporal Gyrus*

1 91 0.02 0.07 58 -12 12

*Uncus*

1 97 0.03 0.11 -35 -10 -28

2 108 0.03 0.08 23 6 -21

*Fusiform Gyrus*

1 111 0.04 0.13 -40 -10 -25

2 2262 0.06 0.15 -35 -44 -13

4 107 0.04 0.14 26 -50 -9

*Inferior Temporal Gyrus*

1 16 0.02 0.05 -38 -9 -28

2 20 0.02 0.04 -44 -43 -17

3 18 0.01 0.02 -60 -53 -4

*Insula*

1 519 0.05 0.15 38 11 -4

2 42 0.02 0.04 -38 -18 -5

3 63 0.02 0.07 47 -12 16

4 131 0.02 0.03 -42 -22 21

*Parahippocampal Gyrus*

1 481 0.04 0.16 -33 -17 -15

2 1268 0.06 0.15 -31 -42 -9

3 64 0.02 0.06 23 6 -17

4 735 0.06 0.16 25 -3 -14

5 151 0.05 0.16 26 -44 -9

6 63 0.04 0.12 -25 -52 3

*Lingual Gyrus*

1 1769 0.06 0.15 -20 -64 0

*Middle Occipital Gyrus*

1 366 0.05 0.15 -27 -66 4

*Middle Temporal Gyrus*

1 13 0.01 0.03 -38 -5 -28

2 2459 0.05 0.14 -56 -48 0

3 316 0.03 0.08 38 -61 26

*Superior Temporal Gyrus*

1 17 0.02 0.05 24 10 -23

2 291 0.05 0.16 37 6 -14

3 17 0.01 0.03 -54 -41 6

4 44 0.03 0.08 39 -52 23

5 76 0.04 0.07 39 -56 27

*Inferior Fronta Gyrus*

1 2519 0.05 0.16 29 17 -14

2 6588 0.04 0.09 54 27 11

3 853 0.04 0.12 -38 36 5

4 36 0.01 0.03 49 2 19

6 42 0.01 0.02 -45 3 32

*Cuneus*

1 162 0.04 0.15 -19 -69 6

*Angular Gyrus*

1 509 0.03 0.08 38 -61 33

*Supramarginal Gyrus*

1 113 0.02 0.08 38 -51 27

2 184 0.01 0.05 -57 -49 36

*Mid Cingulate Gyrus*

1 3170 0.03 0.07 6 -2 35

*Inferior Parietal Lobule*

1 491 0.04 0.10 -43 -26 25

2 2237 0.02 0.05 -50 -46 44

3 221 0.03 0.08 37 -59 39

4 101 0.01 0.03 -30 -51 45

*Precuneus*

1 310 0.02 0.07 33 -62 38

2 85 0.01 0.02 -27 -52 48

3 15 0.01 0.03 -21 -77 46

4 98 0.01 0.03 -23 -70 50

5 65 0.01 0.01 -12 -70 51

*Superior Parietal Lobule*

1 975 0.01 0.03 -29 -58 49

2 92 0.02 0.05 -41 -58 50

3 258 0.01 0.01 -12 -65 55

***Middle Frontal Gyrus***

2 23 0.02 0.05 24 29 -12

3 112 0.04 0.12 -37 44 -3

5 43 0.01 0.01 44 40 -3

7 1066 0.08 0.12 -35 45 10

8 607 0.07 0.09 44 46 8

9 32 0.02 0.06 58 35 14

**10 1770 0.04 0.09 53 28 24**

11 140 0.02 0.04 57 3 39

12 132 0.01 0.02 33 -11 44

*Paracentral Lobule*

1 90 0.01 0.01 1 -12 43

*Postcentral Gyrus*

1 1057 0.05 0.11 54 -12 18

2 497 0.04 0.10 -41 -18 26

3 1019 0.03 0.06 56 -17 43

4 975 0.02 0.04 -48 -15 45

5 320 0.01 0.02 45 -18 54

*Precentral Gyrus*

1 13 0.01 0.01 58 16 9

2 935 0.04 0.11 54 -6 16

4 24 0.01 0.01 -39 -14 27

6 2495 0.02 0.04 -45 -8 43

7 2040 0.03 0.06 57 -6 38

8 1527 0.01 0.02 41 -12 51

*Superior Frontal Gyrus*

1 18 0.02 0.05 -27 44 8

2 51 0.07 0.12 -31 47 14

*Medial Frontal Gyrus*

1 10 0.01 0.02 16 14 -16

*Tuber of Vermis*

1 41 0.01 0.03 0 -72 -23

*Declive of Vermis*

1 648 0.10 0.18 0 -72 -17

*Culmen of Vermis*

1 252 0.07 0.16 1 -64 -7

*Cerebellar Tonsil*

1 16 0.19 0.19 23 -39 -30

*Fastigium*

1 12 0.02 0.03 10 -50 -22

2 308 0.12 0.18 -7 -54 -21

*Nodule*

1 163 0.10 0.19 10 -50 -28

2 405 0.08 0.18 -5 -57 -26

*Uvula*

1 76 0.02 0.07 -8 -63 -27

3 113 0.08 0.18 15 -79 -25

4 12 0.01 0.02 -11 -80 -25

*Pyramis*

1 586 0.06 0.18 -7 -72 -25

2 221 0.06 0.18 9 -78 -25

*Declive*

1 2747 0.09 0.18 -4 -65 -16

2 869 0.08 0.18 11 -78 -20

3 445 0.06 0.19 22 -56 -14

4 105 0.02 0.07 -25 -52 -13

*Culmen*

1 2151 0.07 0.19 21 -45 -18

2 459 0.05 0.15 -40 -41 -22

3 4633 0.08 0.18 -3 -54 -9

5 573 0.05 0.14 -23 -47 -14

*Cerebellar Lingual*

1 402 0.05 0.17 -2 -45 -15

2 3 0.01 0.01 6 -48 -12

*Lentiform Nucleus*

1 14 0.01 0.01 19 13 -10

2 898 0.06 0.16 25 -2 -4

3 15 0.01 0.02 -31 -17 -4

*Claustrum*

1 105 0.04 0.13 33 3 -5

2 51 0.05 0.13 -36 -18 -4

*Thalamus*

1 298 0.03 0.11 17 -29 10

*Caudate*

1 12 0.03 0.04 -34 -15 -9

2 24 0.03 0.05 -34 -25 -5

3 19 0.02 0.03 28 -37 7

4 20 0.02 0.04 25 -36 10

5 25 0.02 0.06 22 -32 14

*Hippocampus*

1 259 0.04 0.14 -32 -19 -12

***Amygdala***

**1 515 0.06 0.16 24 -4 -13**

*CaudateTail*

1 20 0.04 0.07 -34 -16 -8

2 30 0.03 0.05 -34 -25 -4

3 20 0.01 0.03 28 -37 7

4 20 0.02 0.04 25 -36 10

5 25 0.02 0.06 22 -32 14

*Dentate*

1 590 0.07 0.19 16 -50 -24

2 249 0.07 0.18 -9 -55 -24

*Pulvinar*

1 348 0.04 0.11 17 -29 11

*Medial Globus Pallidus*

1 34 0.02 0.05 19 -6 -5

*Lateral Globus Pallidus*

1 247 0.05 0.15 22 -3 -5

*Putamen*

1 14 0.01 0.01 19 13 -10

2 638 0.06 0.16 27 -1 -3

3 20 0.01 0.02 -31 -18 -4

*Brodmann Areas*

*BA1*

2 110 0.04 0.06 55 -16 45

3 72 0.01 0.02 -53 -16 46

*BA2*

2 138 0.02 0.06 55 -21 45

*BA3*

2 381 0.03 0.06 55 -15 42

3 580 0.02 0.04 -49 -15 45

4 206 0.01 0.02 44 -18 54

*BA4*

1 126 0.03 0.07 57 -4 17

3 267 0.03 0.06 56 -14 40

4 603 0.03 0.04 -46 -12 45

5 33 0.02 0.06 55 -6 44

6 435 0.01 0.02 41 -14 52

*BA6*

1 28 0.02 0.02 55 -3 12

3 58 0.02 0.04 51 -3 21

5 368 0.01 0.04 -45 -2 34

6 765 0.03 0.06 59 -3 38

7 36 0.02 0.03 57 -2 33

8 255 0.01 0.02 42 -7 51

10 227 0.02 0.04 -45 -4 49

12 27 0.01 0.01 43 -12 58

*BA7*

1 563 0.01 0.03 -27 -59 49

2 14 0.01 0.01 -21 -76 46

3 72 0.02 0.05 -41 -58 50

5 210 0.01 0.01 -12 -66 54

*BA9*

1 925 0.05 0.09 59 15 26

3 16 0.01 0.01 -45 4 32

*BA10*

1 61 0.08 0.12 -40 47 0

2 47 0.03 0.08 56 40 -1

3 85 0.07 0.09 44 47 2

5 293 0.09 0.12 -38 46 10

6 143 0.05 0.09 44 45 10

*BA11*

4 25 0.03 0.08 24 29 -12

*BA13*

1 515 0.08 0.16 36 12 -9

2 20 0.05 0.08 18 14 -13

3 18 0.02 0.06 50 -10 13

5 80 0.03 0.07 -43 -25 21

*BA18*

1 196 0.03 0.12 -16 -68 -2

2 70 0.02 0.05 -21 -55 3

*BA19*

1 21 0.03 0.06 25 -52 -9

2 338 0.04 0.14 -27 -49 -5

3 12 0.01 0.01 -28 -67 -7

5 458 0.07 0.15 -21 -63 0

8 45 0.02 0.04 34 -63 39

*BA20*

1 292 0.05 0.16 -40 -13 -22

2 337 0.07 0.15 -39 -34 -17

*BA21*

2 475 0.05 0.13 -62 -49 -2

3 24 0.04 0.08 -53 -44 6

*BA22*

1 168 0.05 0.14 -55 -43 3

*BA24*

1 839 0.03 0.07 7 -3 35

2 17 0.01 0.03 14 14 33

3 527 0.03 0.07 -3 -9 38

*BA25*

1 6 0.01 0.02 15 13 -15

*BA28*

1 58 0.03 0.06 24 6 -21

*BA29*

1 15 0.03 0.05 13 -43 7

*BA30*

1 25 0.04 0.07 -25 -53 4

4 132 0.06 0.15 -19 -62 6

*BA31*

1 24 0.01 0.01 5 -13 43

2 24 0.01 0.01 -2 -12 43

*BA32*

1 18 0.01 0.02 15 17 33

2 117 0.02 0.06 14 12 34

*BA34*

1 189 0.04 0.13 23 4 -15

*BA36*

1 335 0.07 0.15 -38 -32 -17

3 35 0.02 0.06 25 -41 -10

4 31 0.01 0.04 -24 -41 -9

*BA37*

1 731 0.06 0.15 -32 -44 -12

2 127 0.05 0.16 27 -47 -9

3 31 0.02 0.03 -58 -49 -6

4 151 0.04 0.13 -58 -55 -2

*BA38*

2 136 0.05 0.16 37 6 -14

*BA39*

1 168 0.04 0.08 39 -57 26

3 233 0.03 0.08 37 -61 36

*BA40*

2 1499 0.02 0.05 -51 -46 43

*BA41*

1 23 0.01 0.03 54 -16 13

*BA42*

1 82 0.03 0.07 60 -10 12

*BA43*

1 502 0.05 0.11 55 -9 14

*BA44*

1 237 0.03 0.08 58 16 12

*BA45*

1 215 0.04 0.09 55 37 2

3 783 0.04 0.09 57 24 9

4 266 0.05 0.09 57 21 20

*BA46*

1 505 0.05 0.09 49 43 6

2 97 0.03 0.12 -44 43 9

3 947 0.03 0.09 55 30 19

4 26 0.03 0.08 -37 36 11

*BA47*

1 1028 0.05 0.15 27 15 -14

2 229 0.03 0.10 25 22 -18

3 39 0.02 0.07 24 29 -10

4 60 0.02 0.07 32 30 -10

6 38 0.01 0.03 57 36 - 2
